# Postoperative complications following mastopexy and reduction mammaplasty in patients with cannabis use disorder: a propensity-matched cohort study^✰^

**DOI:** 10.1016/j.jpra.2026.08.016

**Published:** 2026-08-19

**Authors:** Neil Upreti, Rishi Chilappa, Sri Tummala, Purushottam Nagarkar

**Affiliations:** a School of Medicine, Stanford University, Stanford, CA, United States; b Department of Plastic Surgery, The University of Texas Southwestern Medical Center, Dallas, TX, United States; c Department of Orthopedic Surgery, Baylor University Medical Center, Dallas, TX, United States

**Keywords:** Cannabis, Marijuana, Breast reduction, Mastopexy, Surgical complications, Propensity score matching

## Abstract

**Purpose:**

Cannabis use disorder (CUD) is increasingly prevalent among surgical patients, yet its association with postoperative complications following breast surgery remains poorly characterized. This study compared rates of surgical site complications and emergency department (ED) utilization after mastopexy and reduction mammaplasty between patients with and without documented CUD.

**Methods:**

A retrospective, propensity-matched cohort study was performed using the TriNetX federated research network. Patients undergoing mastopexy or reduction mammaplasty with documented CUD within 3 years before surgery (cannabis cohort) were matched 1:1 to patients without CUD (control cohort) on age, sex, race, ethnicity, comorbidities, and procedure type. Patients with cocaine or methamphetamine use were excluded from both cohorts. Primary outcomes included 90-day rates of surgical site infection (SSI), wound dehiscence, and seroma or hematoma, as well as 30-day ED visits. Risk ratios (RRs) with 95% confidence intervals (CIs) were calculated.

**Results:**

Among 2444 matched patients (1222 per group), CUD was associated with higher rates of surgical site infection (3.8%vs 2.0%; RR, 1.84; *P* = 0.011), seroma or hematoma (3.6%vs 2.0%; RR, 1.83; *P* = 0.014), and 30-day ED visits (14.2%vs 9.6%; RR, 1.48; *P* < 0.001). Wound dehiscence rates did not differ significantly (5.6%vs 4.2%; RR, 1.35; *P* = 0.092). Causal attribution of outcomes to the index procedure could not be established.

**Conclusions:**

CUD was associated with modestly increased rates of wound complications and ED utilization following mastopexy and reduction mammaplasty. While absolute risk differences were small, these findings may inform preoperative counseling and postoperative monitoring in this population.

## Introduction

Mastopexy and reduction mammaplasty are among the most commonly performed breast procedures in plastic surgery, with high rates of patient satisfaction and well-established safety profiles.^1^, ^2^, ^3^ Despite their overall safety, these procedures involve extensive tissue dissection, undermining, and wound closure under tension, creating conditions in which patient-level factors may influence healing and complication risk.^3^ Identifying modifiable or counseling-relevant risk factors remains important for optimizing perioperative outcomes.

Cannabis is the most widely used federally illicit substance in the United States, and its use has increased substantially following the expansion of state legalization policies.^4^^,^^5^ Among surgical populations, the prevalence of cannabis use disorder (CUD) has risen in parallel, raising questions about its potential effects on postoperative outcomes.^6^^,^^7^ Preclinical and clinical evidence suggests that cannabis may impair multiple aspects of wound healing: cannabinoid receptor activation has been associated with suppression of inflammatory cell migration, reduced fibroblast proliferation, and impaired angiogenesis.^8^^,^^9^ Systemically, cannabis use has been linked to airway hyperreactivity, increased sympathetic tone, and altered immune function, each of which may contribute to perioperative risk.^10^, ^11^, ^12^

Prior investigations of cannabis use and surgical outcomes have largely focused on orthopedic, abdominal, and cardiothoracic procedures, with findings that range from increased complication rates to no significant differences.^6^^,^^7^^,^^13^ Studies specific to plastic surgery are limited. A small number of retrospective analyses have examined cannabis use in the context of breast reconstruction, abdominoplasty, and cosmetic surgery broadly, but findings have been inconsistent and often confounded by concurrent substance use.^14^, ^15^, ^16^, ^17^, ^18^ Critically, no large-scale study has examined the association between CUD and postoperative complications following mastopexy or reduction mammaplasty.

Mastopexy and reduction mammaplasty serve as useful clinical models for examining the influence of CUD on wound healing. Both procedures involve extensive subdermal dissection, long suture lines, and tissue rearrangement under tension—features that make wound complications a clinically relevant endpoint. Furthermore, because these procedures are elective, they are less likely to be confounded by the acute physiologic derangements associated with trauma or oncologic surgery.

The purpose of this study was to compare postoperative complications following mastopexy and reduction mammaplasty between patients with documented CUD and those without, examining differences in surgical site infection, wound dehiscence, seroma or hematoma formation, and emergency department utilization. We hypothesized that CUD would be associated with higher rates of these postoperative events.

## Materials and methods

### Study design and reporting standards

This retrospective, propensity-matched cohort study was designed and reported in accordance with the Strengthening the Reporting of Observational Studies in Epidemiology (STROBE) guidelines for cohort studies.

### Data source

Data were obtained from the TriNetX Research Network (TriNetX, Cambridge, MA), a federated electronic health record–based platform that aggregates de-identified patient data from participating healthcare organizations. At the time of analysis, the network included records from 44 healthcare organizations, encompassing both academic medical centers and community-based practices. All data were de-identified in accordance with the Health Insurance Portability and Accountability Act Privacy Rule. Because the study involved secondary analysis of de-identified data without direct patient interaction, it was considered exempt from institutional review board approval.

### Patient selection

Patients who underwent mastopexy or reduction mammaplasty were identified using Current Procedural Terminology (CPT) codes 19,316 (mastopexy) and 19,318 (breast reduction). Patients were excluded if they had any documented diagnosis of cocaine-related disorders, positive methamphetamine laboratory results, or methamphetamine medication orders. These exclusions were applied to isolate the effect of cannabis from other illicit substance use (Table 3).

### Cohort definition

The cannabis cohort comprised patients with documentation of cannabis-related disorders in the three years prior to the index procedure. The control cohort comprised patients without any documented cannabis-related diagnosis codes.

### Propensity score matching

Propensity scores were estimated using logistic regression within the TriNetX analytics platform. Patients in the cannabis cohort were matched 1:1 to patients in the control cohort using a greedy nearest-neighbor algorithm without replacement with a default caliper of 0.1 pooled standard deviations of the logit of the propensity score.

Covariates selected for matching included age at index procedure, sex, race, ethnicity, tobacco use, type 2 diabetes mellitus, alcohol-related disorders, obesity, anxiety, depression, and procedure type (mastopexy vs breast reduction). Balance was assessed using standardized mean differences (SMDs), with values <0.1 considered adequate. Several potentially relevant variables—including body mass index subclass, prior surgical history, hormonal therapy use, and anesthesia type—could not be considered in matching, because these variables were not available within the TriNetX platform.

### Outcomes

Primary outcomes defined a priori included surgical site infection (SSI), wound dehiscence, and seroma or hematoma, identified using ICD-10 and CPT codes within a 90-day follow-up window (Table 3). ED visits within 30 days of the index procedure were assessed as an additional outcome using CPT code 1013,711 (Emergency Department Services). The TriNetX platform identifies diagnostic and procedural codes within the specified time window but cannot confirm a direct temporal or causal relationship between the index procedure and a subsequent event. Patients with historical diagnoses matching the outcome codes (eg, a prior wound dehiscence or ED visit predating the index procedure) were not excluded from either cohort; however, only events occurring within the prespecified post-operative follow-up window were counted as study outcomes.

### Statistical analysis

Absolute event rates were calculated and compared between cohorts. Effect estimates were reported as risk ratios (RRs) with 95% confidence intervals (CIs) generated by the platform’s built-in Z test module. Numbers needed to harm (NNH) were calculated as 1 divided by the absolute risk difference for significant outcomes. Statistical significance was set at *P* < 0.05 (2-sided).

## Results

### Cohort characteristics

**Fig. 1:**
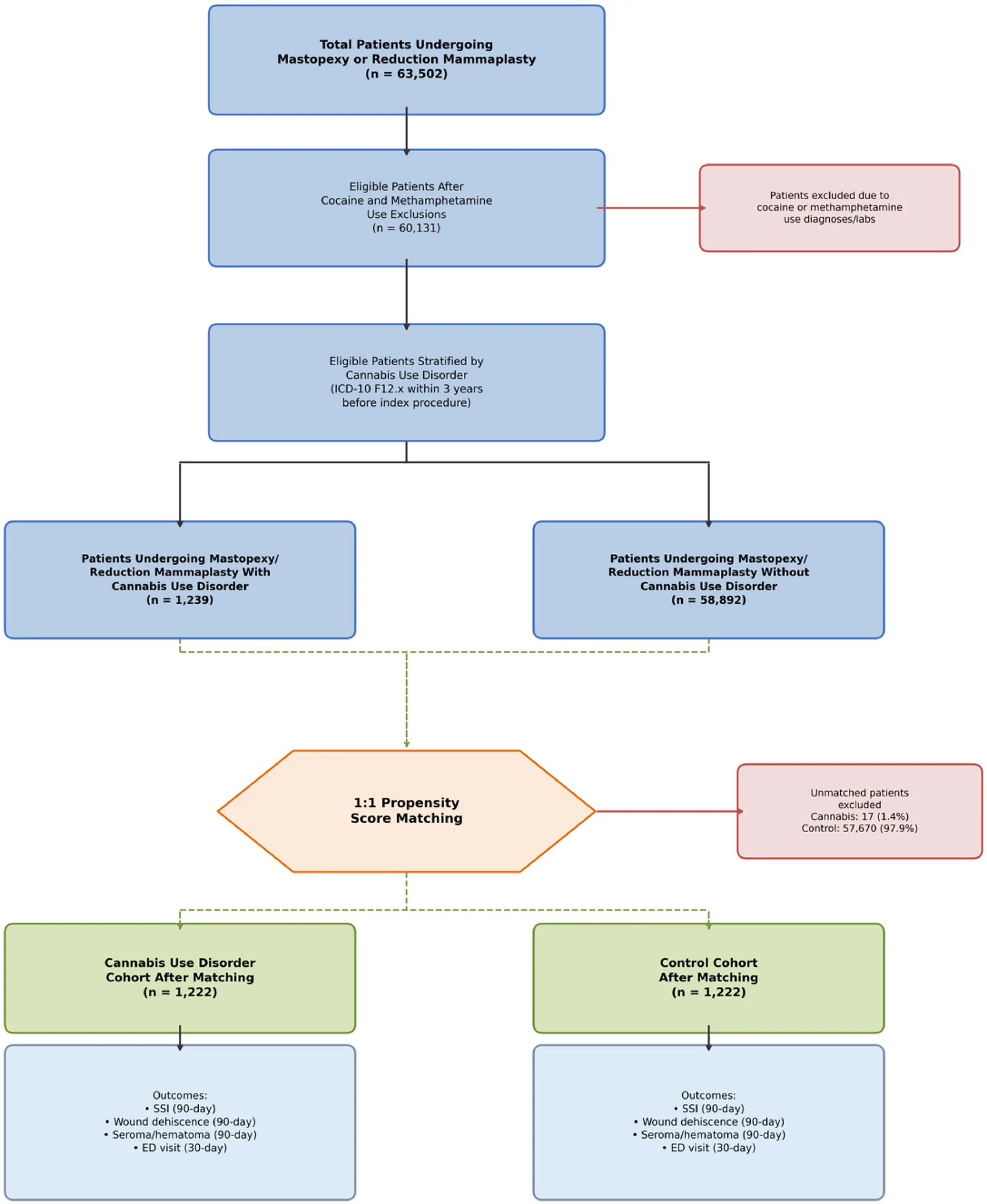
Patient selection flowchart. 63,502 patients underwent mastopexy or reduction mammaplasty. After exclusion of patients with cocaine or methamphetamine use, 1239 met criteria for the cannabis cohort and 58,892 were classified as controls. After 1:1 propensity score matching, each cohort had 1222 patients.

After excluding patients with cocaine or methamphetamine use, there were 60,131 patients who underwent mastopexy or reduction mammaplasty (Fig. 1). 1239 (2.1%) had documented CUD within 3 years before surgery and 58,892 (97.9%) had no cannabis-related diagnoses. Before matching, patients with CUD were substantially younger (mean age at index, 34.2 vs 43.1 years; SMD, 0.637), more likely to be Black or African American (41.6%vs 26.0%; SMD, 0.333), and had markedly higher rates of anxiety (71.6%vs 39.4%; SMD, 0.686), depression (62.1%vs 30.2%; SMD, 0.674), alcohol-related disorders (17.9%vs 2.7%; SMD, 0.515), tobacco use (16.1%vs 3.5%; SMD, 0.433), and overweight or obesity (61.7%vs 44.5%; SMD, 0.350) (Table 1). After 1:1 matching, there were 1222 patients per cohort (98.6% of the cannabis cohort was successfully matched), and all covariates achieved SMDs below 0.1, indicating adequate balance (Table 2).

**Table 1:** Baseline characteristics before propensity score matching.

Characteristic	Cannabis (n = 1239)	Control (n = 58,892)	P Value	SMD
Age in years at index, mean ± SD	34.2 ± 12.9	43.1 ± 15.0	<.001	0.637
Female, n (%)	1193 (96.3)	57,315 (98.7)	<.001	0.153
Male, n (%)	44 (3.6)	749 (1.3)	<.001	0.148
White, n (%)	561 (45.3)	34,143 (58.8)	<.001	0.273
Black or African American, n (%)	515 (41.6)	15,122 (26.0)	<.001	0.333
Asian, n (%)	11 (0.9)	800 (1.4)	.142	0.046
Hispanic or Latino, n (%)	123 (9.9)	5397 (9.3)	.446	0.022
Type 2 diabetes mellitus, n (%)	187 (15.1)	7778 (13.4)	.082	0.049
Tobacco use, n (%)	200 (16.1)	2055 (3.5)	<.001	0.433
Alcohol-related disorders, n (%)	222 (17.9)	1592 (2.7)	<.001	0.515
Overweight and obesity, n (%)	764 (61.7)	25,826 (44.5)	<.001	0.350
Anxiety disorders, n (%)	887 (71.6)	22,868 (39.4)	<.001	0.685
Depressive episode, n (%)	769 (62.1)	17,557 (30.2)	<.001	0.674
Mastopexy, n (%)	141 (11.4)	11,815 (20.3)	<.001	0.247
Breast reduction, n (%)	1113 (89.8)	47,291 (81.4)	<.001	0.241

SMD indicates standardized mean difference. Race and ethnicity categories are not mutually exclusive and may not sum to 100%.

**Table 2:** Baseline characteristics after propensity score matching.

Characteristic	Cannabis (n = 1222)	Control (n = 1222)	P Value	SMD
Age at index, mean ± SD	34.3 ± 12.9	34.1 ± 12.6	.697	0.016
Female, n (%)	1180 (96.6)	1185 (97.0)	.567	0.023
Male, n (%)	40 (3.3)	36 (2.9)	.641	0.019
White, n (%)	555 (45.4)	573 (46.9)	.465	0.030
Black or African American, n (%)	507 (41.5)	517 (42.3)	.682	0.017
Asian, n (%)	11 (0.9)	10 (0.8)	.827	0.009
Hispanic or Latino, n (%)	121 (9.9)	117 (9.6)	.732	0.014
Type 2 diabetes mellitus, n (%)	181 (14.8)	182 (14.9)	.955	0.002
Tobacco use, n (%)	185 (15.1)	175 (14.3)	.568	0.023
Alcohol-related disorders, n (%)	205 (16.8)	200 (16.4)	.786	0.011
Overweight and obesity, n (%)	752 (61.5)	739 (60.5)	.590	0.022
Anxiety disorders, n (%)	871 (71.3)	880 (72.0)	.686	0.016
Depressive episode, n (%)	752 (61.5)	744 (60.9)	.740	0.013
Mastopexy, n (%)	141 (11.5)	127 (10.4)	.365	0.037
Breast reduction, n (%)	1096 (89.7)	1110 (90.8)	.339	0.039

SMD indicates standardized mean difference. All SMDs were <0.1 after matching, indicating adequate covariate balance across cohorts.

**Table 3:** CPT, ICD-10, and LOINC codes used for patient identification, exclusions, and outcome definition.

Category	Code	Description
Procedures	CPT 19,316	Mastopexy
	CPT 19,318	Breast reduction
Cannabis Cohort	ICD-10 F12.x	Cannabis-related disorders (within 3 years before index)
Matching Covariates	ICD-10 E11.x	Type 2 diabetes mellitus
	ICD-10 F17.x	Tobacco use
	ICD-10 F10.x	Alcohol-related disorders
	ICD-10 E66.x	Overweight and obesity
	ICD-10 F40.x, F41.x	Anxiety disorders
	ICD-10 F32.x, F33.x	Depressive episode
Exclusions (Cocaine)	ICD-10 F14.x	Cocaine-related disorders
Exclusions (Methamphetamine)	LOINC 3778–8	Methamphetamine [Mass/volume] in Serum or Plasma
	LOINC 3777–0	Methamphetamine [Presence] in Serum or Plasma
	LOINC 16,235-4	Methamphetamine [Mass/volume] in Urine by Confirmatory method
	LOINC 19,555-2	Methamphetamine [Presence] in Urine by Confirmatory method
	LOINC 3780–4	Methamphetamine [Mass/volume] in Urine
	LOINC 3779–6	Methamphetamine [Presence] in Urine
	LOINC 19,554-5	Methamphetamine [Presence] in Urine by Screen method
	RxNorm 6816	Methamphetamine (medication order)
Outcomes: SSI	ICD-10 T81.4x	Infection following a procedure
Outcomes: Dehiscence	ICD-10 T81.3x	Disruption of wound, not elsewhere classified
Outcomes: Seroma/Hematoma	ICD-10 L76.x	Postprocedural hematoma and seroma of skin/subcutaneous tissue
	ICD-10 M96.841	Postprocedural hematoma of musculoskeletal structure
	CPT 10,140	Incision and drainage of hematoma, seroma, or fluid collection
Outcomes: ED Visit	CPT 1013,711	Emergency Department Services

CPT indicates Current Procedural Terminology; ICD-10, International Classification of Diseases, Tenth Revision; LOINC, Logical Observation Identifiers Names and Codes; SSI, surgical site infection; ED, emergency department.

### Primary outcomes

**Fig. 2:**
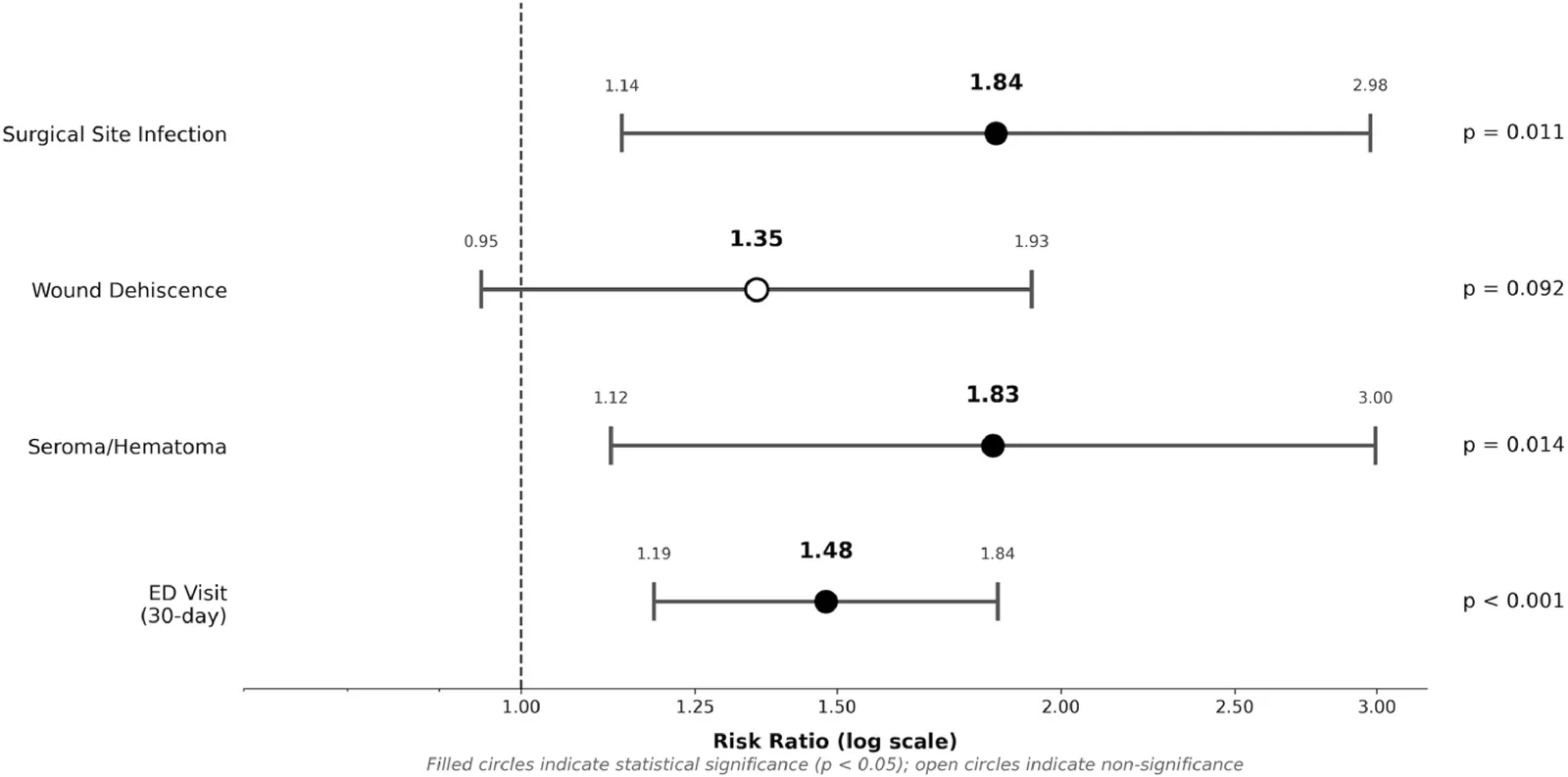
Forest plot comparing the risk of postoperative complications between patients with cannabis use disorder and matched controls without cannabis use disorder following mastopexy or reduction mammaplasty. Risk ratios with 95% CIs are shown for wound outcomes within the 90-day follow-up window and ED visits within the 30-day window after propensity score matching (n = 1222 per cohort). Filled circles indicate statistically significant associations (*P* < 0.05); the open circle indicates a nonsignificant association.

Postoperative outcomes are summarized in Table 4 and Fig. 2.

**Table 4:** Postoperative outcomes after propensity score matching.

Outcome	Cannabis n (%)	Control n (%)	RR (95% CI)	P Value	NNH
SSI†	46 (3.8)	25 (2.0)	1.84 (1.14–2.98)	.011	56
Dehiscence†	69 (5.6)	51 (4.2)	1.35 (0.95–1.93)	.092	—
Seroma/Hematoma†	44 (3.6)	24 (2.0)	1.83 (1.12–3.00)	.014	63
ED Visit‡	173 (14.2)	117 (9.6)	1.48 (1.19–1.84)	<.001	22

CI indicates confidence interval; ED, emergency department; NNH, number needed to harm; RR, risk ratio; SSI, surgical site infection. Dash indicates NNH not applicable for nonsignificant findings. † 90-day follow-up window. ‡ 30-day follow-up window. No correction for multiple comparisons was applied.

***Local wound complications.*** Surgical site infection occurred in 3.8% of the cannabis cohort compared with 2.0% of controls (RR, 1.84; 95% CI, 1.14–2.98; *P* = 0.011; NNH 56). Seroma or hematoma rates were similarly elevated in the cannabis cohort (3.6%vs 2.0%; RR, 1.83; 95% CI, 1.12–3.00; *P* = 0.014; NNH 63). Wound dehiscence was numerically more common in the cannabis cohort but did not reach statistical significance (5.6%vs 4.2%; RR, 1.35; 95% CI, 0.95–1.93; *P* = 0.092).

***Emergency department utilization.*** ED visits were documented in 14.2% of the cannabis cohort versus 9.6% of the control cohort within 30 days of surgery (RR, 1.48; 95% CI, 1.19–1.84; *P* < 0.001; NNH 22).

## Discussion

In this large, propensity-matched analysis of patients undergoing mastopexy or reduction mammaplasty, cannabis use disorder was associated with modestly higher rates of postoperative surgical site infection, seroma or hematoma, and 30-day ED visits compared with matched controls. Wound dehiscence was numerically more frequent in the cannabis cohort but did not reach statistical significance. Although statistically significant, absolute risk differences for wound complications remained small (<2 percentage points), with the largest absolute difference observed for ED utilization (4.6 percentage points).

To contextualize these findings, we compared complication rates in our matched control cohort with those reported in larger published studies of comparable elective breast procedures. In the most directly comparable analysis, Habarth-Morales et al. examined 62,639 patients undergoing reduction mammaplasty in the same TriNetX database, with a matched non-TXA control cohort exhibiting rates of 3.5% for postoperative infection and 2.0% for combined seroma or hematoma during the follow-up window.^19^ In our matched control cohort, the seroma or hematoma rate (2.0%) closely matched their 2.0% benchmark, while our SSI rate (2.0%) was somewhat lower than their 3.5% figure—a difference plausibly explained by the broader infection coding strategy used in the prior analysis and by variability in postoperative follow-up between the two TriNetX runs. Together, these comparisons suggest that the matched control group is broadly representative of the general TriNetX reduction mammaplasty population, although exact rates differ across studies. Wound dehiscence rates in our cohort (4.2% in controls; 5.6% in the cannabis cohort) were higher than the 0.8% to 0.9% reported in NSQIP-based analyses. ,^20^ a discrepancy attributable in part to differences in follow-up window and ascertainment methodology (administrative ICD-10 coding vs trained surgical clinical reviewers). Within this context, the modestly elevated rates observed in the cannabis cohort point to a real, if small, association between CUD and postoperative wound complications, rather than an artifact of an atypical comparison group. Surgeons evaluating patients with documented cannabis use should consider this association alongside the broader constellation of demographic and comorbidity factors (younger age, higher rates of psychiatric comorbidity, tobacco use, and alcohol-related disorders) that frequently co-occur with CUD and may compound perioperative risk.

The pattern of wound complications observed in the cannabis cohort warrants careful interpretation. Endocannabinoid system activation has been shown to modulate inflammatory cell chemotaxis, reduce fibroblast proliferation, and impair neovascularization—processes critical to incisional wound repair.^8^^,^^9^^,^^11^ These mechanisms could plausibly contribute to the elevated rates of surgical site infection and seroma or hematoma observed in our cannabis cohort, as both outcomes are sensitive to impaired early inflammatory signaling, microvascular function, and local immune competence. Cannabis-associated immunomodulation, in particular, has been linked to altered macrophage activity and reduced clearance of bacterial colonization, which may partly account for the elevated infection risk. Wound dehiscence, by contrast, did not reach statistical significance despite a numerically higher rate in the cannabis cohort and a point estimate (RR, 1.35) consistent with the magnitude of association seen for the other wound outcomes. Several factors may contribute to this finding. Dehiscence depends not only on biologic healing but also on mechanical factors such as closure technique, wound tension, and patient activity, which may dilute a cannabis-specific signal. The 95% confidence interval for the dehiscence risk ratio (0.95–1.93) is also compatible with a clinically meaningful effect, raising the possibility that this analysis was underpowered to detect a true difference. Larger or pooled cohorts will be needed to determine whether the apparent dichotomy between dehiscence and the other wound outcomes is real or a chance result of limited sample size.

The elevated 30-day ED visit rate in the cannabis cohort warrants careful interpretation. The TriNetX platform does not provide the reason for an ED visit; therefore, these visits may not be related to the index procedure. Furthermore, even if these ED visits were related to the surgery, ED utilization is a composite measure influenced by multiple factors beyond surgical complications, including pain management patterns, psychiatric comorbidity, health literacy, and access to outpatient follow-up.^21^ Although propensity score matching balanced psychiatric comorbidities (anxiety, depression) and substance use variables, unmeasured differences in healthcare utilization patterns, social support, and pain perception may contribute to the observed difference.

Several additional methodological considerations temper interpretation:

1. CUD was identified using ICD-10 diagnostic codes, which capture clinically documented use disorders but likely underestimate the true prevalence of cannabis use. Patients who use cannabis recreationally without receiving a formal diagnosis would be misclassified into the control group, biasing results toward the null.
2. Despite propensity score matching on key covariates, several important confounders were unavailable, including body mass index subclass, smoking quantity, timing and dose of cannabis exposure relative to surgery, anesthesia type, and surgeon volume. The distinction between active use at the time of surgery and remote historical use is particularly relevant, as the 3-year lookback window may include patients who were actually free of cannabis exposure within several months of the index procedure.^22^^,^^23^
3. Outcome ascertainment relied on ICD-10 and CPT codes and was not restricted to incident diagnoses. Therefore, some captured events may represent preexisting conditions documented during follow-up encounters.
4. Finally, TriNetX captures data only from participating organizations, and complications managed at nonparticipating institutions would not be recorded.

From a pragmatic standpoint, these results may inform but should not restrict perioperative decision-making. The absolute risk of wound complications likely does not justify limiting access to surgery for patients with CUD, as mastopexy and reduction mammaplasty remain broadly safe across both groups studied. Instead, these data may support enhanced preoperative counseling regarding the modestly elevated risk of wound complications.^17^^,^^18^ Surgeons may consider discussing cannabis use during preoperative assessment and advising cessation before elective procedures, though evidence-based guidelines on optimal cessation intervals are currently lacking.^24^^,^^25^ Future prospective studies with granular temporal data on cannabis exposure, standardized outcome adjudication, and shorter follow-up windows are needed to clarify whether the observed associations reflect causal effects of cannabis on wound healing or confounding by unmeasured patient characteristics.

## Ethical approval

Not required.

## Institutional review board statement

This study involved secondary analysis of de-identified data from the TriNetX Research Network and was exempt from institutional review board approval in accordance with the HIPAA Privacy Rule.

## Declaration of competing interest

None declared.

## Data Availability

The data that support the findings of this study are available from TriNetX, LLC (Cambridge, MA), but restrictions apply to the availability of these data, which were used under license for the current study, and so are not publicly available. Data access may be requested through TriNetX for researchers at participating healthcare organizations.

## Appendix Supplementary materials

Image, application 1
