# Supplementary material for: Postoperative complications following mastopexy and reduction mammaplasty in patients with cannabis use disorder: a propensity-matched cohort study
Source: JPRAS Open. 2026 Aug 19;52:138–45. doi: 10.1016/j.jpra.2026.08.016 (PMC13560023; doi:10.1016/j.jpra.2026.08.016)
Supplement: Supplementary file 1 [file mmc1.docx]

**STROBE Statement—Checklist of items that should be included in reports of cohort studies**

*Manuscript: Postoperative Complications Following Mastopexy and Reduction Mammaplasty in Patients With Cannabis Use Disorder: A Propensity-Matched Cohort Study*

Study design: retrospective, propensity-matched cohort study.

| **Topic** | **Item** | **Recommendation** | **Reported in manuscript (section / location)** |
| --- | --- | --- | --- |
| Title and abstract | 1 | (a) Indicate the study's design with a commonly used term in the title or the abstract  (b) Provide in the abstract an informative and balanced summary of what was done and what was found | (a) Title (“A Propensity-Matched Cohort Study”); Abstract, Methods (“retrospective, propensity-matched cohort study”)  (b) Abstract (Purpose, Methods, Results, Conclusions) |
| **Introduction** | | | |
| Background/rationale | 2 | Explain the scientific background and rationale for the investigation being reported | Introduction, paragraphs 1–4 |
| Objectives | 3 | State specific objectives, including any prespecified hypotheses | Introduction, final paragraph (stated purpose and a priori hypothesis) |
| **Methods** | | | |
| Study design | 4 | Present key elements of study design early in the paper | Methods, “Study Design and Reporting Standards”; Abstract, Methods |
| Setting | 5 | Describe the setting, locations, and relevant dates, including periods of recruitment, exposure, follow-up, and data collection | Methods, “Data Source” (TriNetX; 44 healthcare organizations); exposure lookback (3 years pre-index) and follow-up windows (30- and 90-day) in “Cohort Definition” and “Outcomes.” Note: specific calendar dates of data extraction not stated. |
| Participants | 6 | (a) Give the eligibility criteria, and the sources and methods of selection of participants; describe methods of follow-up  (b) For matched studies, give matching criteria and number of exposed and unexposed | (a) Methods, “Patient Selection” and “Cohort Definition”; Table 3 (codes); Figure 1  (b) Methods, “Propensity Score Matching”; Results (1,222 per cohort); Figure 1; Tables 1–2 |
| Variables | 7 | Clearly define all outcomes, exposures, predictors, potential confounders, and effect modifiers; give diagnostic criteria, if applicable | Methods, “Cohort Definition,” “Propensity Score Matching” (covariates), “Outcomes”; Table 3 (CPT/ICD-10/LOINC definitions) |
| Data sources/measurement | 8 | For each variable of interest, give sources of data and details of methods of assessment (measurement); describe comparability of assessment methods if there is more than one group | Methods, “Data Source” and “Outcomes”; Table 3. Same coding applied identically to both cohorts via the TriNetX platform. |
| Bias | 9 | Describe any efforts to address potential sources of bias | Methods (exclusion of cocaine/methamphetamine use to isolate cannabis; 1:1 propensity score matching); Discussion, limitations (non-differential misclassification biasing toward the null) |
| Study size | 10 | Explain how the study size was arrived at | Results / Figure 1: all eligible matched patients were included; no a priori sample-size calculation was performed (fixed by available cohort). |
| Quantitative variables | 11 | Explain how quantitative variables were handled in the analyses; if applicable, describe which groupings were chosen and why | Methods, “Propensity Score Matching” (age modeled as a continuous covariate) and “Statistical Analysis” (absolute event rates; risk ratios) |
| Statistical methods | 12 | (a) Describe all statistical methods, including those used to control for confounding  (b) Describe any methods used to examine subgroups and interactions  (c) Explain how missing data were addressed  (d) If applicable, explain how loss to follow-up was addressed  (e) Describe any sensitivity analyses | (a) Methods, “Propensity Score Matching” (logistic regression, greedy nearest-neighbor 1:1, caliper 0.1; SMDs) and “Statistical Analysis” (RRs, 95% CIs, Z test, NNH)  (b) No subgroup or interaction analyses were performed  (c) Not specifically addressed (federated de-identified platform); noted as a limitation  (d) Fixed 30-/90-day windows used; unrecorded events at non-participating institutions noted as a limitation  (e) None performed |
| **Results** | | | |
| Participants | 13 | (a) Report numbers of individuals at each stage—eg numbers potentially eligible, examined for eligibility, confirmed eligible, included, completing follow-up, and analysed  (b) Give reasons for non-participation at each stage  (c) Consider use of a flow diagram | (a) Results, “Cohort Characteristics”; Figure 1  (b) Figure 1 (cocaine/methamphetamine exclusions; unmatched patients excluded)  (c) Figure 1 (patient selection flowchart) |
| Descriptive data | 14 | (a) Give characteristics of study participants and information on exposures and potential confounders  (b) Indicate number of participants with missing data for each variable of interest  (c) Summarise follow-up time (eg, average and total amount) | (a) Results; Tables 1–2 (pre- and post-matching characteristics)  (b) Not reported (platform-level de-identified data)  (c) Fixed follow-up windows (30- and 90-day) reported in Methods/Outcomes; per-patient follow-up time not applicable |
| Outcome data | 15 | Report numbers of outcome events or summary measures over time | Results, “Primary Outcomes”; Table 4; Figure 2 |
| Main results | 16 | (a) Give unadjusted estimates and, if applicable, confounder-adjusted estimates and their precision (eg, 95% CI); make clear which confounders were adjusted for and why  (b) Report category boundaries when continuous variables were categorized  (c) If relevant, consider translating estimates of relative risk into absolute risk for a meaningful time period | (a) Results; Table 4; Figure 2 (RRs with 95% CIs after 1:1 matching; matched covariates listed in Methods)  (b) Not applicable (no continuous variables were categorized)  (c) Results / Table 4 (absolute event rates and NNH); Discussion |
| Other analyses | 17 | Report other analyses done—eg analyses of subgroups and interactions, and sensitivity analyses | NNH calculation (Methods/Results); benchmarking of control rates against prior TriNetX and NSQIP cohorts (Discussion). No subgroup or sensitivity analyses performed. |
| **Discussion** | | | |
| Key results | 18 | Summarise key results with reference to study objectives | Discussion, opening paragraph |
| Limitations | 19 | Discuss limitations of the study, taking into account sources of potential bias or imprecision; discuss both direction and magnitude of any potential bias | Discussion (bulleted limitations: coding-based misclassification biasing toward null, unmeasured confounders, exposure timing, prevalent vs incident coding, non-participating institutions; underpowering for dehiscence; ED-visit interpretation) |
| Interpretation | 20 | Give a cautious overall interpretation of results considering objectives, limitations, multiplicity of analyses, results from similar studies, and other relevant evidence | Discussion (biologic plausibility; comparison with prior studies; cautious framing; note that no correction for multiple comparisons was applied—Table 4 footnote) |
| Generalisability | 21 | Discuss the generalisability (external validity) of the study results | Discussion (representativeness of the matched control cohort vs published benchmarks; limitation that TriNetX captures only participating organizations) |
| **Other information** | | | |
| Funding | 22 | Give the source of funding and the role of the funders for the present study and, if applicable, for the original study on which the present article is based | Title page and end-of-text statement (“Funding: None”) |

*Note: An Explanation and Elaboration article discusses each checklist item and gives methodological background and published examples of transparent reporting. The STROBE checklist is best used in conjunction with this article (freely available at www.strobe-statement.org).*
